# Role of a complex of two proteins in alleviating sodium ion stress in an economic crop

**DOI:** 10.1371/journal.pone.0242221

**Published:** 2020-11-20

**Authors:** Jie Yang, Mingyu Liu

**Affiliations:** 1 Capital University of Economics and Business, Beijing, China; 2 Beijing Forestry University, Beijing, China; Hainan University, CHINA

## Abstract

An economically valuable woody plant species tree bean (*Cajanus cajan (L*.*) Millsp*.) is predominantly cultivated in tropical and subtropical areas and is regarded as an important food legume (or pulse) crop that is facing serious sodium ion stress. NAM (N-acetyl-5-methoxytryptamine) has been implicated in abiotic and biotic stress tolerance in plants. However, the role of NAM in sodium ion stress tolerance has not been determined. In this study, the effect of NAM was investigated in the economically valuable woody plant species, challenged with stress at 40 mM sodium ion for 3 days. NAM-treated plants (200 μM) had significantly higher fresh weight, average root length, significantly reduced cell size, increased cell number, and increased cytoskeleton filaments in single cells. The expression pattern of one of 10 Tree bean Dynamic Balance Movement Related Protein (TbDMP), TbDMP was consistent with the sodium ion-stress alleviation by NAM. Using TbDMP as bait, Dynamic Balance Movement Related Kinase Protein (TbDBK) was determined to interact with TbDMP by screening the tree bean root cDNA library in yeast. Biochemical experiments showed that NAM enhanced the interaction between the two proteins which promoted resist sodium ion stress resistance. This study provides evidence of a pathway through which the skeleton participates in NAM signaling.

## Introduction

**Fig 1:**
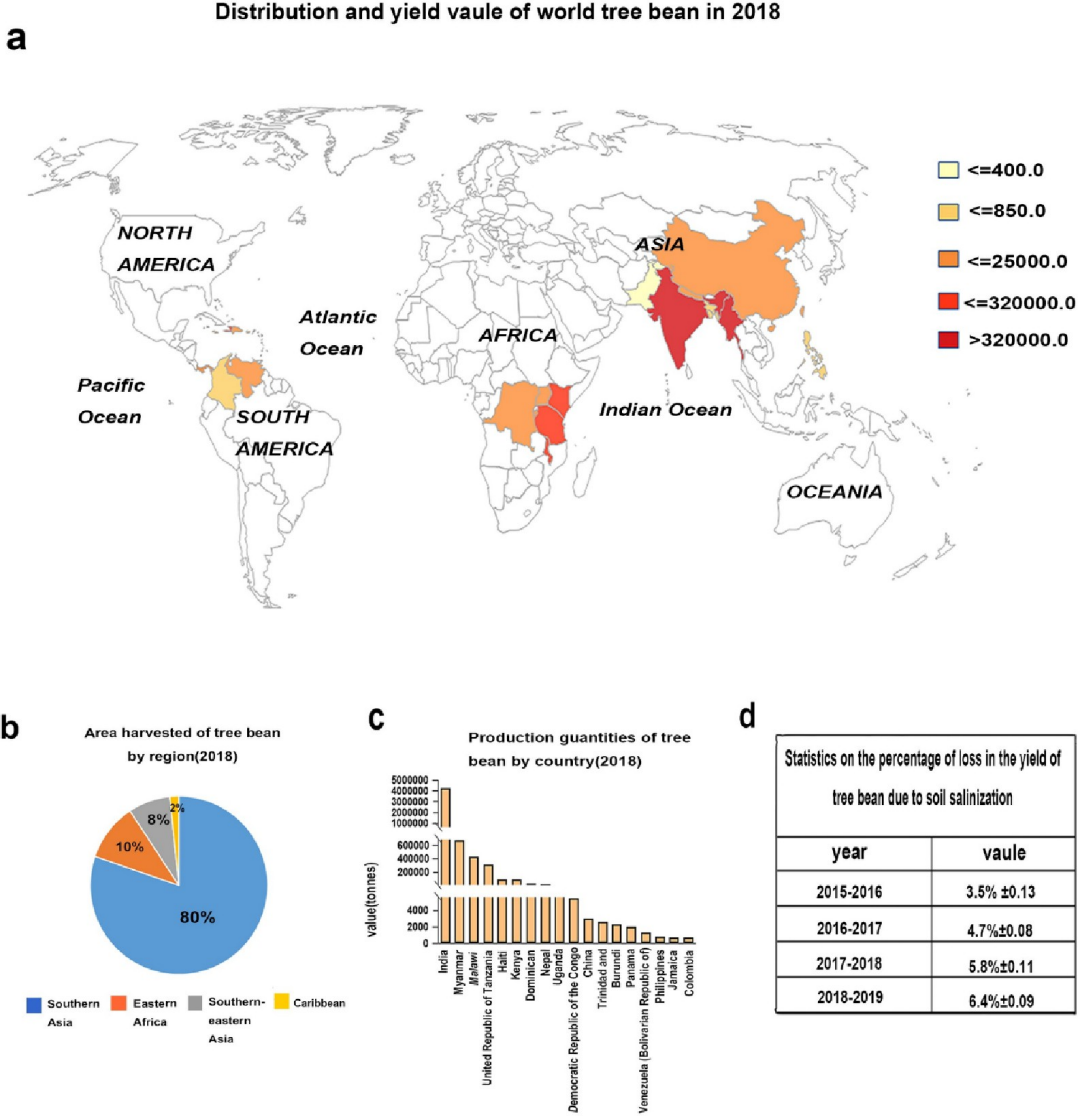
The distribution, output value and the loss due to sodium ion stress of the tree bean in the world. a. Tree bean cultivation area and national production. b. Proportion of planting area by region. c. The ranking of the output value of each country, the bar represents each country, and the vertical axis represents the output value of the current year. d. Proportion of loss of tree bean production caused by sodium ion stress in the past five years.

Tree bean is an edible bean crop with the sixth highest production among crops worldwide; it is grown primarily in the tropical and subtropical regions of the world and has a planting area of 7 million hectares and a production volume of 6 million tons (FAOSTAT 2018). Tree bean is a major staple food for more than one billion people and an important cash crops that has abundant nutritional and medicinal value [1]. In 2018, the global harvest area of tree bean was approximately 7 million hectares, which was concentrated primarily in Asia (87% of the world harvested area), with India, Myanmar, China, Bangladesh, and Pakistan representing the major producers. The yield varies significantly by region, as represented by various colors in Fig 1A. Dark red corresponds to the regions with the highest yield (>320 kilotons). The yield of the red area is less than or equal to 320 kilotons, the yield of the orange area is less than 25 kilotons, and the yields of the yellow and light yellow areas are less than 850 *tons* and 400 *tons*, respectively (Fig 1A).

South Asia is the largest planting area and accounts for 80% of the total planting area, and East Africa accounts for 10% of the plant area. Southeast Asia accounts for only approximately 10% of the area, and the Caribbean accounts for 2% of the area (Fig 1B).

From a production perspective, India is the world’s largest producer of tree bean at 4.3 million *tons*, which accounts for 72% of the world production, and it is followed by Myanmar and Malawi, which output 0.67 million tons and 0.43 million tons, respectively, accounting for 11% and 7.2% of the world production (Fig 1C).

However, over the past five years, the percentage of loss in the yield of tree bean due to soil salinization has increased annually as follows: 3.5%±0.13 in 2015–2016, 4.7%±0.08 in 2016–2017, 5.8%±0.11 in 2017–2018 and 6.4%±0.09 in 2018–2019 (Fig 1D).

Since most areas of tree bean growth are arid and semiarid, high soil salinity is a main problem. Sodium ion stress can reduce the yield and quality and influence the metabolism by destroying the water potential, producing ionic toxicity, and decreasing cell membrane integrity and function and the absorption of essential nutrients, which causes yield reduction and diminished quality annually [2]. Therefore, it is important to identify the sodium ion tolerance-related genes and proteins and to establish a strategy to alleviate sodium ion stress in tree bean.

NAM is a versatile molecule that is widely used in animals. NAM is seldom used in plants; however, it has been shown to be an abiotic antistress agent [3]. In animal disease and cancer studies, NAM inhibits cancer cell invasion and metastasis via phosphorylation of kinases-regulated and microtubule organization and ultimately inhibits cellular skeleton dynamics to suppress the spread of cancer cells [4, 5]. NAM has typical immunoregulatory and circadian activity in animals that is mediated by cytoskeleton rearrangement [6]. Plants are sessile organisms that have to cope with various environmental changes during their life cycles. In the process of evolution, plants developed a mechanism to maintain a low cytoplasmic sodium ion content to avoid the adverse effects of sodium ion on plant growth and development. Sodium ion is mainly manifested by osmotic stress, which leads to intracellular ion imbalance. Cell signaling pathways are triggered by sodium ion stress, and dynamic changes in the cytoskeleton are generated by cellular responses, such as a transient increase in cytosolic free Ca^2+^ levels [7]. The cytoskeletal elements are composed of cytoskeleton microfilaments, microtubules and intermediate filaments and are extensively integrated to play a key role in cell growth, division, and sodium ion stress [8]. The actin cytoskeleton plays an important role in reorganization in response to stresses and cellular stimuli [8]. Microfilaments are highly dynamic in organisms and undergo rapid recombination and transformation during growth. This process is assisted by a number of participating Dynamic Balance Movement Related Protein (DMP), such as ADF, Villin, Profilin, Fimbrin and Capping Proteins [9]. DMP proteins play a role in regulation of the kinetics and reorganization of filaments by binding to globular and filamentous cytoskeletons [10]. The DMP family of proteins is an important family of Dynamic Balance Movement Protein that regulate cytoskeleton filament dynamics [11]. In animals and plants, DMP activity is regulated by the phosphorylation state of the serine residue of a protein that regulates cytoskeleton disassembly. The phosphorylation of DMP by calcium-stimulated protein kinase always determines the DMP activity in plant cells [12]. The sodium ion exchanger is involved in the control of cellular sodium ion homeostasis during Sodium ion tolerance and this process is regulated by a protein kinase and a calcium-binding protein in *Arabidopsis thaliana* [13]. In animals, DMP is known as cofilin; and itis phosphorylated on Ser-3 in animals or the equivalent Ser-6 in plants. The site of phosphorylation of DMP by CDPK kinase is Ser-6 in *Zea mays L* [14]. Extracellular stimuli, cytoskeleton rearrangement, and cell activities largely determine the Ser-3 phosphorylation levels; thus, phosphorylation plays a key role in the regulation of cofilin activity. Based on an amino acid sequence similarity search and tertiary structure prediction, the functional similarity between Ser-6 in plant DMP and Ser-3 in animal cofilin was studied [15]. In animals, many kinase proteins phosphorylate and deactivate DMP; however, animal kinases do not phosphorylate plant DMPs and plant kinase does not phosphorylate animal DMPs [16]. Previous studies have shown that the sodium ion stress response involves the SOS pathway. The SOS signaling system refers to a system that regulates the signal transduction pathway of ion balance inside and outside the cell. It mediates the efflux of Na^+^ in the cell and the regional distribution into the vacuole under sodium ion stress, regulates ion homeostasis and improves sodium ion tolerance via the regulation of MF organization [17], including SOS3-like calcium binding protein 1, SOS2-like protein kinase 5, and the plasma membrane H^+^-ATPase [18]. sodium ion stress induces the accumulation of reactive oxygen species (ROS), such as hydrogen peroxide (H_2_O_2_), superoxide anion (O_2_^-^), and hydroxyl radical (•OH), which can cause programmed cell death [19]. ROS production may lead to Ca^2+^ influx, which promotes more ROS production. Under Sodium ion stress, SOS can form a negative feedback loop to regulate the Ca^2+^ signature in response to Sodium ion stress by phosphorylating and repressing the Ca^2+^-permeable transporter.

In a further study, we speculate that kinase proteins may represent initial response regulators of a signal transduction cascade and participate in regulation of the cytoskeleton in response to environmental stimulation and plant development. In the present study, NAM was shown to alleviate sodium ion stress by enhancing the TbDMP-TbDBK interaction and rearranging cytoskeleton in source of tree bean production data.

## Materials and methods

### Source of tree bean data

Data on the harvested area, regional area proportions and national production value of tree bean were obtained from the Food and Agriculture Organization in 2018 from an agricultural statistical database that is available for download((http://www.fao.org/home/en/). Data on sodium ion-stressed disasters of tree bean were collected during field research from 2015 to 2019. The tree bean is sodium ion-sensitive and widely cultivated globally.

### Plant material, growth conditions and growth parameter measurements

Seeds of tree bean which are sodium ion sensitive, were obtained from the Northeast Forestry University of China. After sterilizing the surface with 0.1% mercury chloride for 5 min, the seeds were washed with sterilized water five times. Afterwards, the seeds were germinated in 10-cm (diameter) × 9-cm (height) pots containing soil and sand at a 3:1 volumetric ratio. The plants were then maintained in a greenhouse at 25°C, The Sodium ion stress treatment was performed with 0, 20, 40, 80 and 100 mM sodium ion and the sodium ion stress-alleviating NAM levels of 0, 50, 100, 200, and 400 μM. The growth parameters, including the shoot height, primary root length, and fresh weight, were measured after a recovery period of 48 h by maintaining the plants under the growth conditions.

### Staining and quantification of cytoskeletons in root cells

Technovit 7100 embedding resin was used for the histological analysis according to Clément et al. [20]. A Leica RM2265 microtome was used to section the embedded tissues at 5-mm thickness. The sections were stained in 0.05% toluidine blue for 15 min and finally examined under a Leica CTR5000B microscope. ImageJ software was used to estimate the cell area, cell number, and cytoskeleton filament number (http://rsb.info.nih.gov/ij/). The root tip region was obtained in the medium and fixed in 350 mM MBS (Sigma-Aldrich; prepared in the germination medium, i.e., MS medium) to stop the reaction. The root tips were then washed with 0.15% Nonidet P-40 in germination medium for 10 min followed by three additional washes for 10 min with 0.08% Nonidet P-40 in TBSS buffer (pH 7.5) (50 mM Tris–HCl, 250 mM sodium ion, and 10% sucrose). Root tips were then stained with 232 nM rhodamine–phalloidin (Sigma-Aldrich) in TBSS with 0.15% Nonidet P-40 at 25°C for 3 h. After three washes with TBSS, stained root tips were visualized using a spinning disc confocal microscope equipped with a 100x oil immersion objective (CSU-X1, Andor, http://www.andor.com/) as described by Yang et al. [21].

### RT-PCR and qRT-PCR analysis

RNA was extracted from the freshly prepared samples using a Direct-zol RNA MiniPrep kit (Zymo Research) according to the manufacturer’s instructions. The quality of the extracted RNA was determined using a spectrophotometer (NanoDrop). RNA was reverse transcribed using a QuantiTect reverse transcription kit (Qiagen). qRT-PCR was performed on an Applied Biosystems 7500 real-time PCR system (Life Technologies) using SYBR Premix Ex Taq II (TaKaRa), as described by Dong et al. [20]. Tree bean cytoskeleton was used as a reference gene for normalization. The primers used in this study are listed in S2 Table.

### Yeast cDNA library preparation and TbDBK screening

We used the full-length CDS of *TbDMP* as a bait to screen a yeast cDNA library prepared from the tree bean roots and the yeast two-hybrid (Y2H) library was constructed according to the Y2H system user manual tree bean. The coding sequence of *TbDMP* was cloned into the pGBKT7 vector as bait. Total RNA was isolated from the root tissue using the CTAB method as previously described. The empty vectors pGBKT7 and pGADT7 empty vectors were used as negative controls, whereas the vectors pGBKT7-p53 and pGADT7-T were used as positive controls.

### BiFC assay

TbDMP, TbDBK, and TbDMP-like were cloned into the pSYPNE and pSYPCE vectors with a YFP conserved domain to generate N-terminal or C-terminal fusion proteins, respectively. The plasmids were introduced into *Agrobacterium tumefaciens* GV3101 and infiltrated into *Nicotiana benthamiana* as previously described [20]. Infected tissues were imaged after 48 h using a Leica SP8 confocal microscope. Immunoblotting was performed to confirm the expression of various fusion proteins using HA (product No. PA1-985, lot No. RI237644) or c-Myc antibody (product no. PA1-981, lot No. SB247983) for the tag at the C-terminus and N-terminus of YFP, respectively (all antibodies from Invitrogen).

### Subcellular colocalization analysis

The *TbDMP-eGFP* and *TbDBK*-mcherry fusion genes were separately cloned into the p-CAMBIA1300 vector under the control of the 35S promoter. Constructs were transformed into *Agrobacterium tumefaciens* strain GV3101.

Agrobacterium cultures grown overnight were centrifuged, washed, and resuspended to an optical density of 0.1–0.2 at 600 nm, as described previously by Yang et al. [11]. Using a 1-mL syringe, the diluted agrobacterial cultures were infiltrated into the leaf tissues of 4- to 5-week-old N. benthamiana plants grown in a greenhouse at 22°C to 24°C. Protein fluorescence was observed 72 h after agroinfiltration. A Leica SP8 confocal microscope and ImageJ software were used to acquire and analyze the images. The green and red fluorescence values along the linear distance were calculated as grayscale values by the Plot Profile tool where 0 is black and 255 is white. Then, using the rectangular tool, the corresponding fluorescence channel was delineated using Plot Profiled. The fluorescence intensity changes and the rectangular range were obtained to derive the data from the image.

This approach was used for XY scanning and Z-axis slicing (XYZ scanning). The XYZ scanning mode is the default acquisition mode, and Z-axis scanning was used to observe the spatial distribution of the target in the sample. After XYZ image acquisition, the image was opened in the 3D viewer of the 3D visualization module. A number of parameters were adjusted in the display mode. The analysis of co-localization was based on Image J software.

### Hairy root transgenic analysis

The *TbDMP*-RNAi and *TbDBK*-RNAi coding sequences were cloned into the pROK2 vector and transformed into the *Agrobacterium rhizogenes* strain K599. Then, the K599 strains harboring the empty vectors or the genes were cultured in 5 mL of YEP liquid medium supplemented with 20 mg/L rifampicin and 50 mg/L kanamycin under shaking (180 rpm) at 28°C for approximately 14 h. Afterwards, the bacterial suspension was centrifuged at 8,000 rpm for 10 min at room temperature and resuspended in MES buffer (10 mM MES-KOH, pH 5.2, 10 mM MgCl_2_ and 100 μM acetosyringone). The solution was injected into healthy and uniform subcultured seedlings of tree bean. After approximately one month of culture of the transgenic hairy roots, the plant tissue was prepared to perform RT-qPCR. We divided the number of withered tree beans by the total amount of tree beans to calculate the average We divided the number of withered tree beans to calculate the average withered amount.

## Results

Globally, an area of nearly 10 million hectares is affected by Sodium ion. Soil salinity remains one of the most serious environmental problems in agriculture and limits agricultural production worldwide [22]. Tree bean is a major grain and legume crop that can withstand Sodium ion stress, and it is widely cultivated in the tropics in saline-alkali areas. Tree bean grows well in barren soil because of its deep root system and abundant lateral roots, which assist in extracting water during Sodium ion periods [23]. Developing an economical and efficient method of promoting Sodium ion stress resistance in tree bean is important for realizing its economic value and requires further exploration.

In this study, the role of melanin in promoting Sodium ion tolerance in tree bean was explored. Experiments showed that melanin can enhance the interaction of two proteins in tree bean to achieve Sodium ion stress resistance and allow tree bean to resist soil salinization in arid and semiarid areas, thereby promoting its economic value.

To determine a suitable sodium ion concentration for the NAM treatment, 5-day-old tree bean seedlings were subjected to various sodium ion concentrations (0, 20, 40, 80 and 100 mM sodium ion) for 3 days. The average root length and fresh weight were measured to select a suitable treatment, which was identified as 40 mM sodium ion (S1A and S1B Fig). Subsequently, the sodium ion-treated seedlings were grown in the presence of various NAM levels (0, 50, 100, 200, and 400 μM) to determine an appropriate level of NAM for sodium ion stress relief (S1C Fig). The fresh weight accumulation was significantly higher in plants treated with 200 μM NAM compared with that in the control; however, higher NAM concentrations significantly inhibited fresh weight gain. Our results indicate that treatment with 200 μM NAM is the most suitable for mode to alleviating sodium ion stress caused by 40 mM sodium ion.

**Fig 2:**
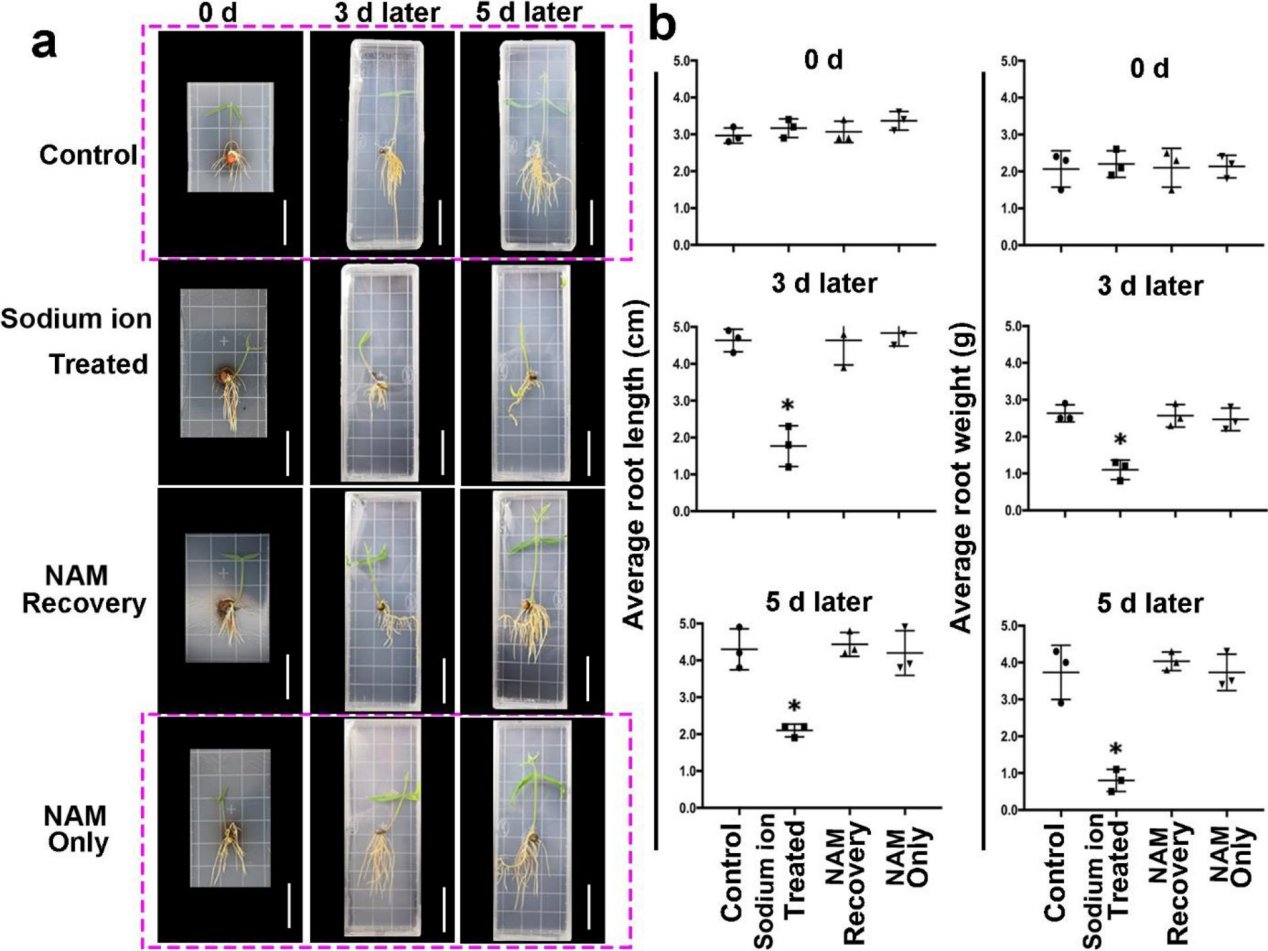
NAM alleviates tree bean growth under sodium ion tress. a. Tree bean seeds grown for 5 days on medium supplemented with control, sodium ion (40 mM) and NAM (200 μM NAM adding in the sodium ion stress) treatment in 0, 3 and 5 DAT (Days After Treatment). b. Effect of the different treatment on (a) average root length and fresh weight of tree bean plant after 2 days of recovery from sodium ion-stress treatment.

Differences in the degree of Sodium ion stress tolerance in 5-day-old seedlings were analyzed to identify the alleviating effect of NAM on tree bean seedlings of various ages. After sodium ion treatment for 3 days, the fresh weight and average root length were significantly lower than those in the zero day group. When NAM was added to the sodium ion stress groups, the phenotypes had been restored in seedlings of both ages. In summary, NAM can alleviate sodium ion stress effectively at day 5 (Fig 2).

**Fig 3:**
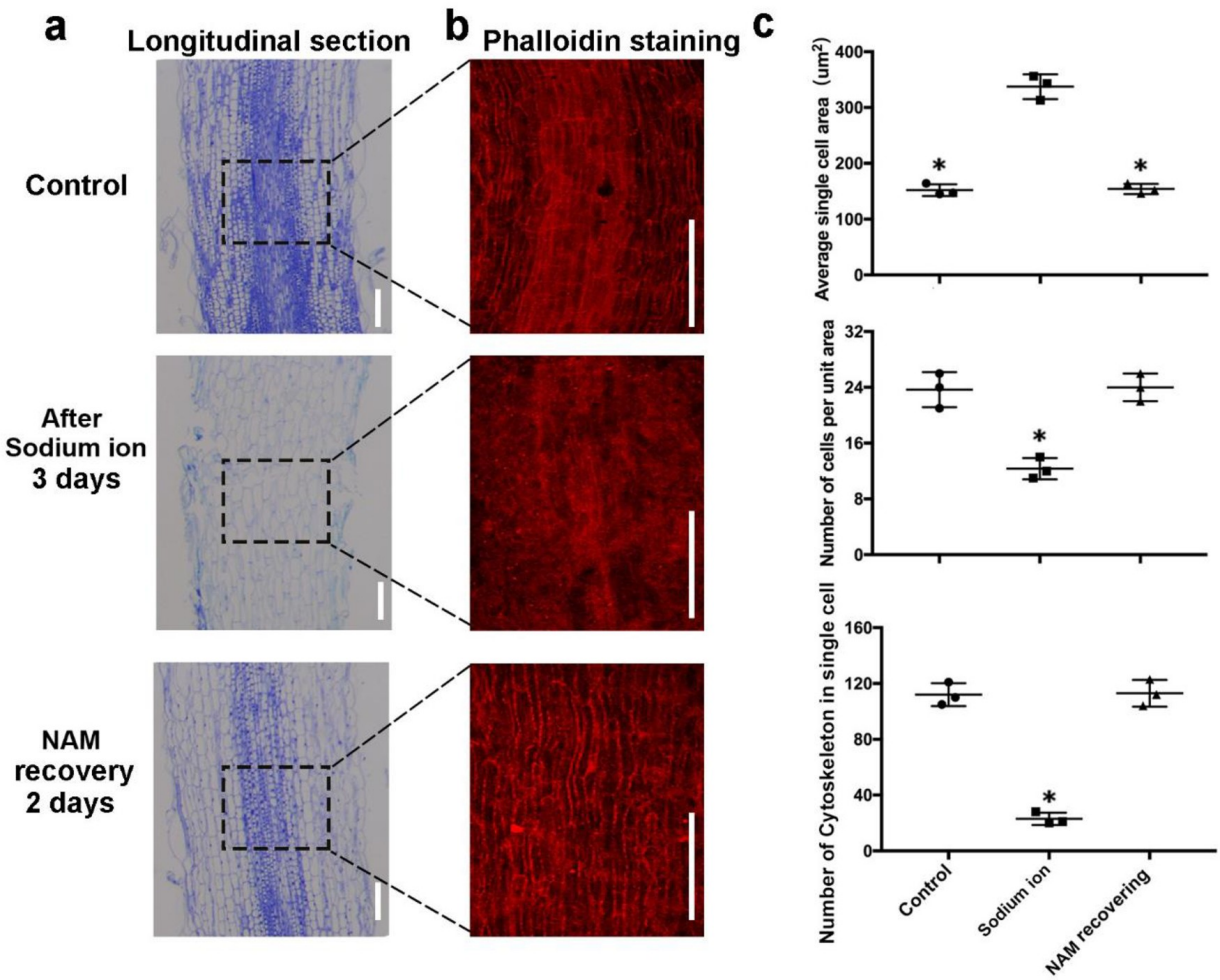
NAM alleviates root microfilament structure. a. Longitudinal section of the root elongation region in the mature zone of the control, Sodium ion and NAM root seedlings. b. Rhodamine-labeled phalloidin staining of roots showing the different Cytoskeleton structures in root cells. c. Differential average single cell area on the outer and inner sides of the root region in (b). Number of cells was counted and their size was measured from ~50 cross sections of the roots in (a). Numbers of Cytoskeleton in a single cell was measured from ~30 Longitudinal sections of the roots in (b). In (c) error bars show the standard error of the mean (SEM; n = three biological replicates). *P< 0.05 (Student’s t-test). Bars = 200 μm.

Numerous reports demonstrated that NAM can alleviate sodium ion stress in plants [24, 25]; however, the involvement of changes in the cytoskeleton and ABP are involved in this process has not been clarified. Initially, we observed the root architecture and arrangement of the root cells in tree bean by microscopy. Longitudinal sections of the roots showed that the cell length on the inner (concave) side of both organs was significantly shorter than that on the outer (convex) side in the sodium ion-treated samples, whereas differences were not observed between the control and NAM recovery groups (Fig 3A). Then, we examined the morphology of the cytoskeleton in the root tip cells, and find that the sodium ion-treated samples had more coarse bundles of cytoskeleton and fewer short cytoskeleton filaments, which appeared in a disordered manner. After alleviating sodium ion stress, NAM was able to significantly reduce the area of a single cell, increase the number of cells and increase the average number of cytoskeleton fibers in a single cell (Fig 3C). Moreover, the NAM treatment apparently recovered the disordered arrangement of cytoskeleton. Thus, NAM can reverse the phenotypic effects of sodium ion stress on cell morphology and cytoskeleton disorder.

**Fig 4:**
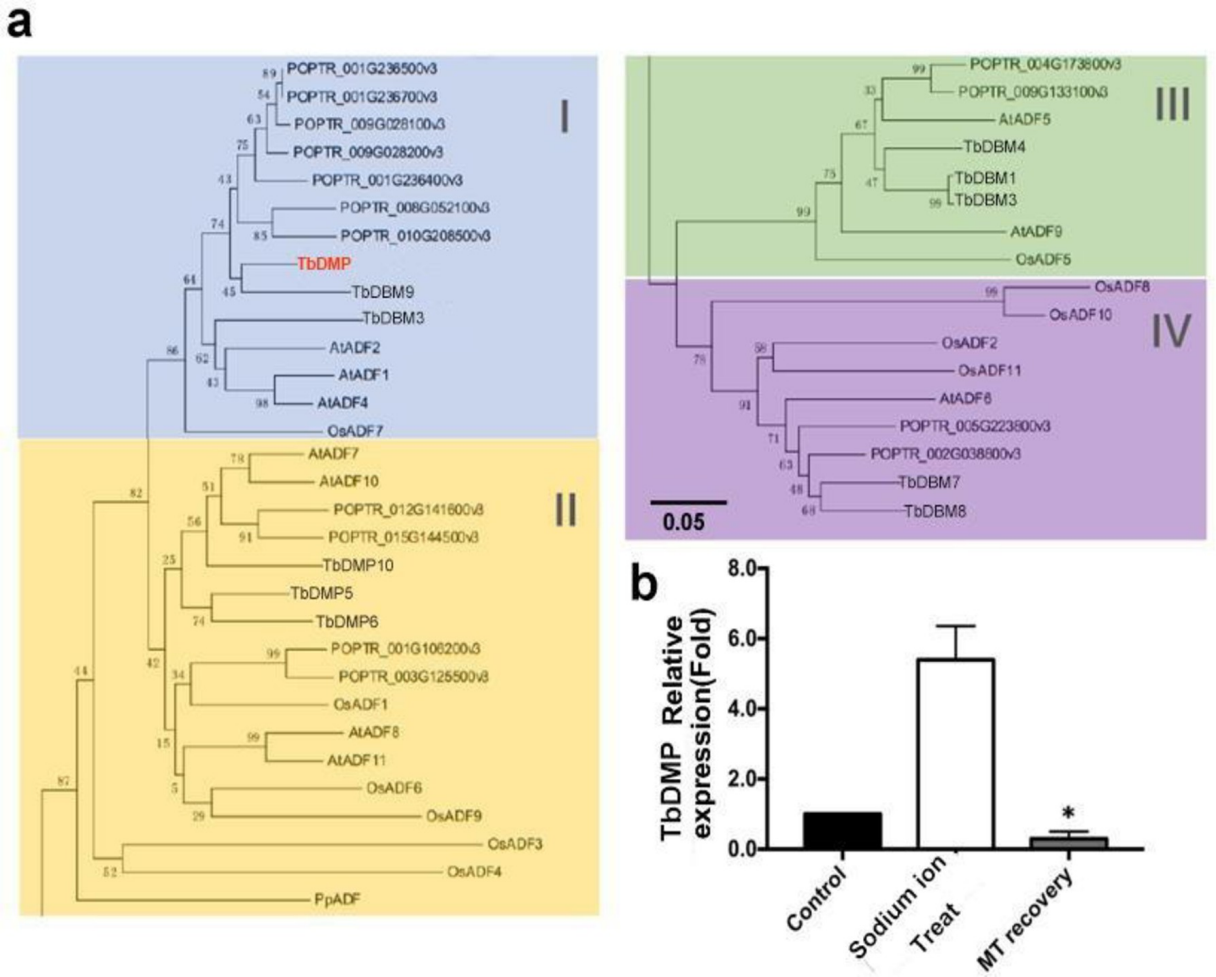
Tissue specific expression analysis of TbDMP genes in tree bean. a. Phylogenetic tree of identified TbDMP proteins. The phylogenetic tree using MEGA 6.0 program. The proteins were classified into four classes, I to IV. The sequences were aligned by ClustalW. The bootstrap values of 1000 replicates were calculated at each node. b. Rt-PCR indicated TbDMP genes expression pattern under control, Sodium ion stress and NAM recovery. Three biological replicates in each group. In (b) error bars show the standard error of the mean (SEM; n = three biological replicates). *P< 0.05 (Student’s t-test).

We hypothesized that the *DMP* gene family in tree bean has an important role in regulating of cytoskeleton filament dynamics and reorganization, which has not been previously reported. To identify the *TbDMP* gens involved in this process, we detected the expression of all *TbDMP* genes under various conditions, and found that there are a total of 10 *TbDMP* genes in tree bean. After the sodium ion treatment, only the relative expression level of a single TbDMP was increased. After sodium ion stress was alleviated viaNAM, the expression of TbDMP was reduced to a level similar to that in the control group (Fig 4B). This result indicates that TbDMPs are involved in the process of sodium ion stress alleviation by NAM.

*TbDMP* was previously shown to function in a complex with other proteins; hence, we searched for potential proteins interacting with TbDMP. The following criteria were used to select suitable proteins: strong interaction with TbDMP as assessed by the Y2H assay high frequency of potential proteins that interact with TbDMP as indicated by Y2H screening and bimolecular fluorescence complementation (BiFC) analysis. Therefore, we used TbDMP from tree bean as bait to screen a pollen yeast cDNA library, and we found the interaction with TbDMP were identified (S2 Table). The disassembling activity of cytoskeleton is influenced by a number of external environments in animals, plants and microorganisms. This process can be completed with the assistance of TbDMP proteins. Furthermore, enhancing TbDMP activity frequently requires assistance of other proteins, such as cytoskeleton-interaction protein-1 and cyclase-associated protein [20, 26, 27]. In A*rabidopsis thaliana*, TbDBK physically interacts with cytoskeleton-depolymerizing factor 4 and inhibits its activity by phosphorylation during cytoskeleton filament disassembly and TbDBK regulates cytoskeleton filament reorganization and stomatal closure, primarily through phosphorylation of TbDMP. Strikingly, TbDBK was found as a potential interaction partner of TbDMP.

**Fig 5:**
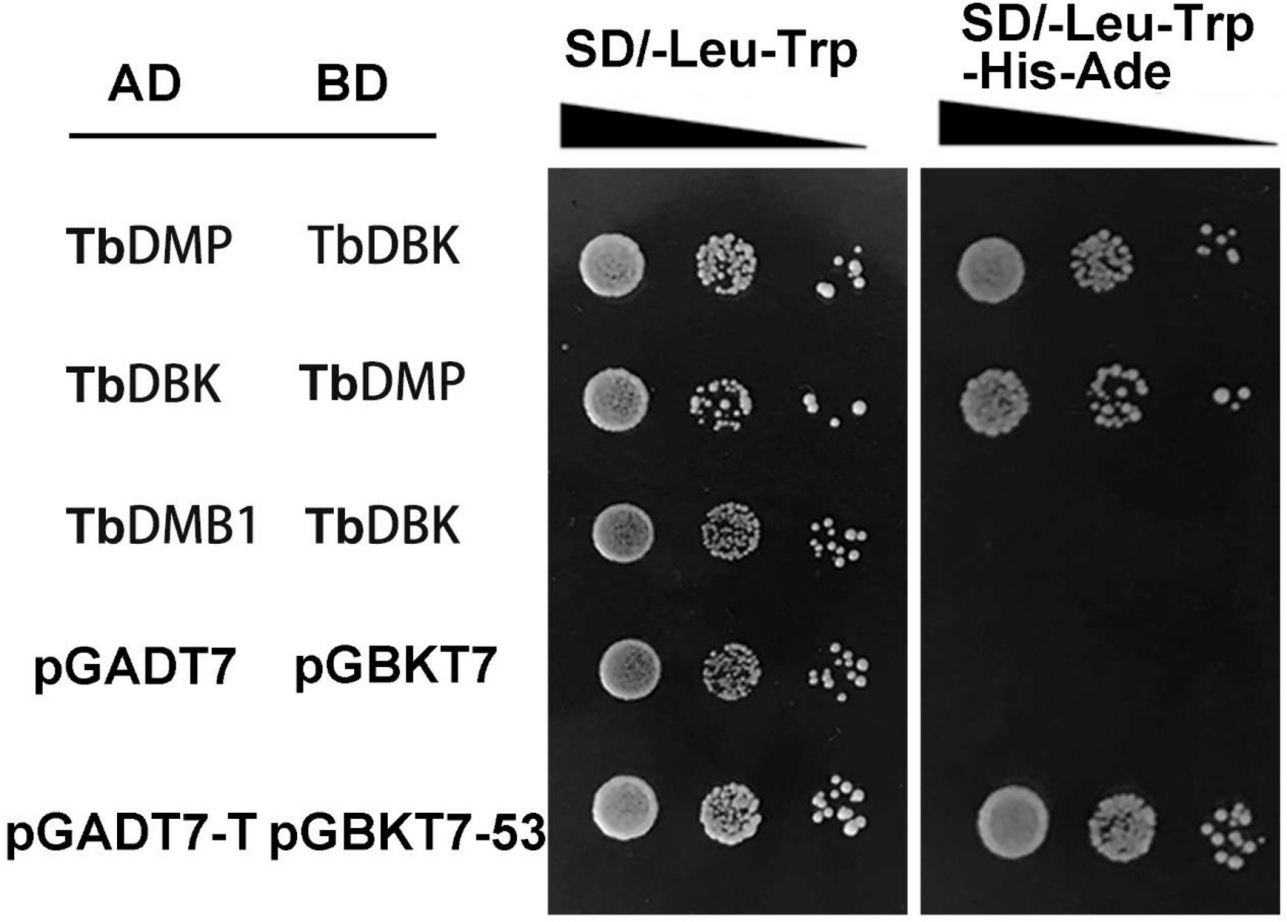
Y2H assay of the Interaction between TbDMP and TbDBK. The pGADT7-T and pGBKT7-p53 pair was used as a positive control. Pairs of AD-TbDBM and BD-TbDBK, and AD-pGADT7 and BD-pGB-KT7 were used as negative controls. Each colony was dissolved in 10 μL sterile water and then diluted to 10^−1^ to 10^−3^. At least three colonies per combination were tested.

**Fig 6:**
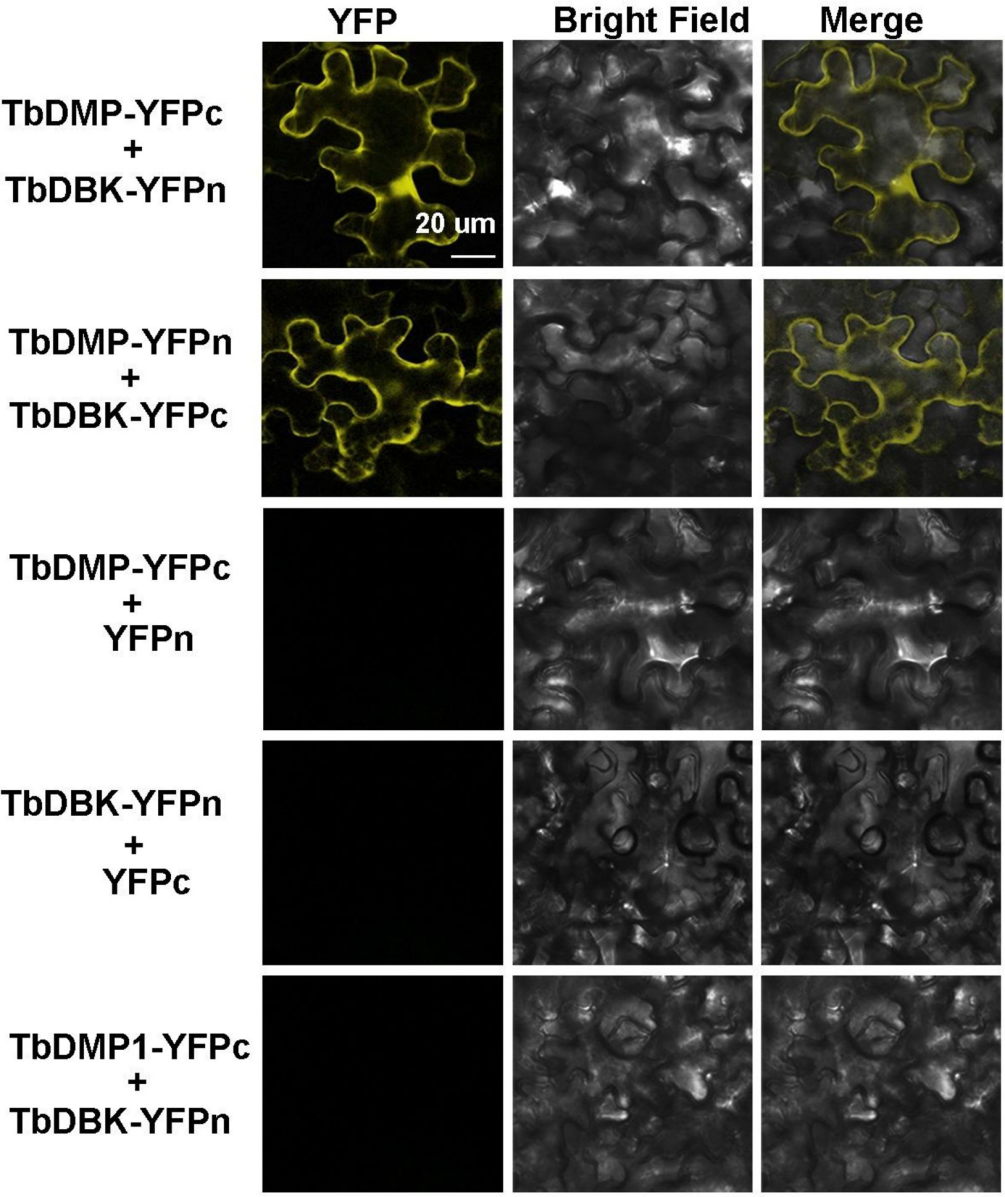
BiFC assay of the interaction between TbDMP and TbDBK in tobacco (*Nicotiana benthamiana*) leaves. TbDMP and TbDBK were introduced into pSYPNE or pSYPCE vectors, respectively, fused with N-terminal or C-terminal YFP. Pairs of TbDMP—YFPc+YFPn, TbDBK -YFPn+YFPc, and TbDBM1-YFPc+ TbDBK -YFPn were used as negative control. Immunoblots on the right side obtained using HA or c-Myc antibody for the tag at C-terminal and N-terminal YFP, respectively, showing the expression of various fusion proteins. Bars = 20 μm.

To further verify the interaction between TbDMP and TbDBK, we transfected the full length CDS of TbDMP and TbDBK into the yeast strain AH109 and found that TbDMP has a strong physical interaction with TbDBK (S3 Table). The interaction was further confirmed by the BiFC assay in tobacco leaves using laser scanning confocal microscopy. A strong YFP fluorescence signal was observed after cotransfection of nYFP-TbDMP and TbDBK-cYFP and TbDMP-cYFP and nYFP-TbDBK, respectively (Fig 5). Western blotting was used to examine the expression of TbDMP and TbDBK in the BiFC assay and TbDMP-like was used as a control (Fig 6). The results indicate that TbDMP and TbDBK interact in the leaf cells. Thus, the findings indicate that TbDMP can physically interact with TbDBK.

**Fig 7:**
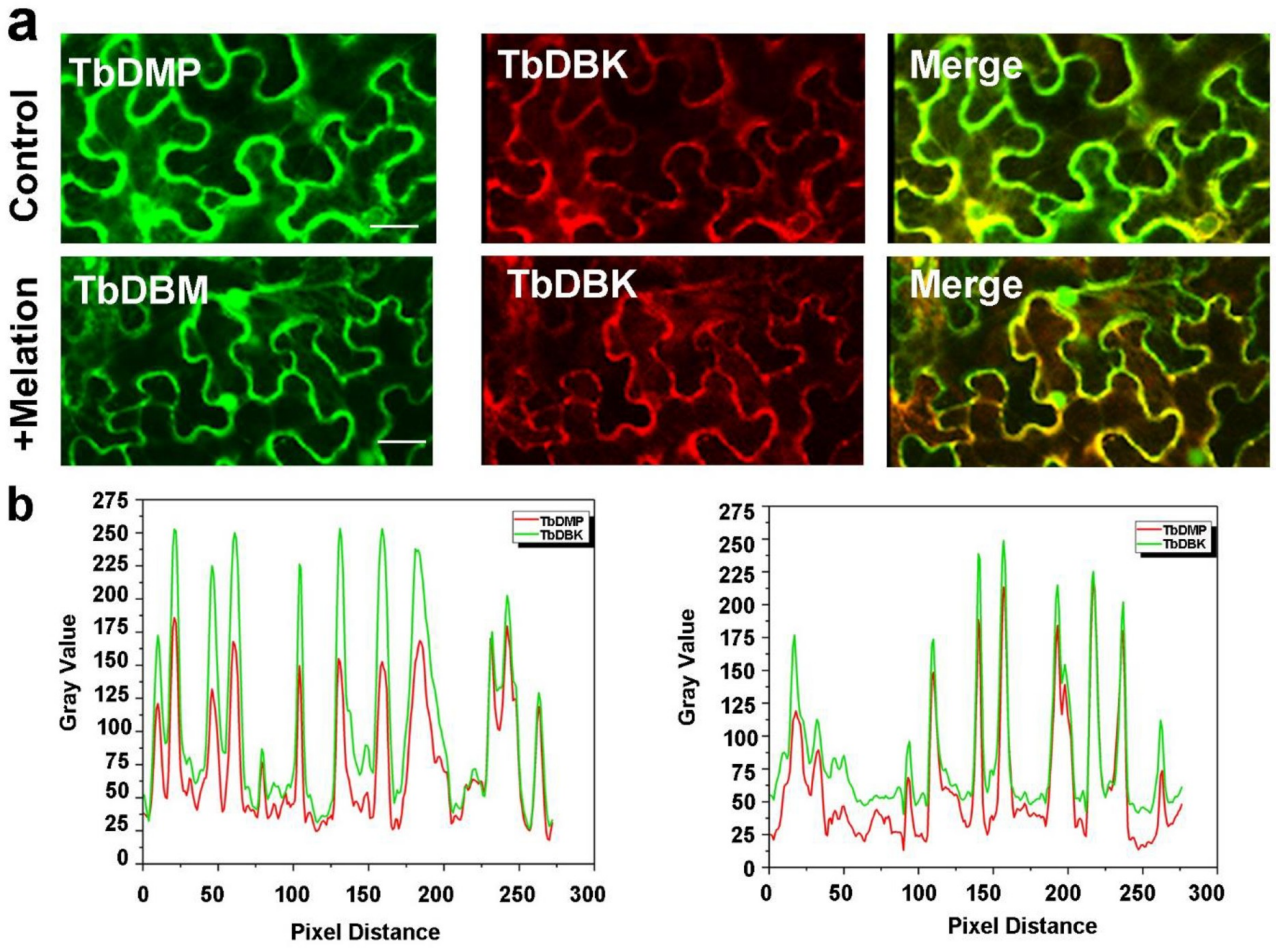
Subcellular colocalization analysis of TbDMP and TbDBK in tobacco (*Nicotiana benthamiana*) leaves. a. TbDMP -GFP and TbDBK -mCherry were transiently co-expressed in *N*. *benthamiana* leaves in control and preying NAM treatment groups. Scale bars = 20 μm. b. Software ImageJ was used to acquire and analysis of co-localization. The gray value of green and red fluorescence along the line distance can be obtained by Plot Profile tool. Colocalization of TbDMP -GFP and TbDBK—m-Cherry in different cells. The experiment was repeated three times.

**Fig 8:**
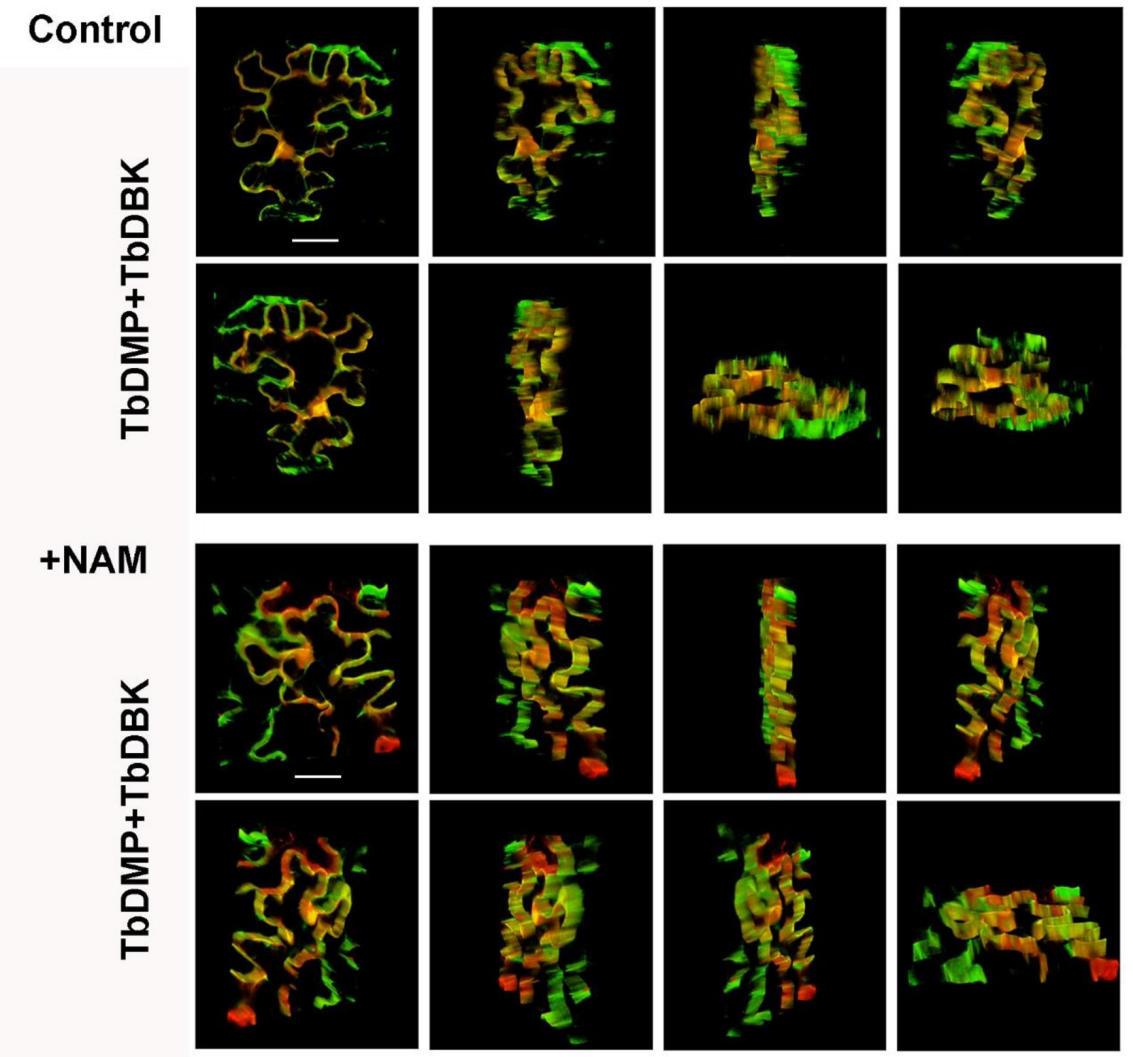
3D subcellular colocalization analysis of TbDMP and TbDBK under NAM. The analysis of 360 degrees in all directions 3D visualization module colocalization.

**Fig 9:**
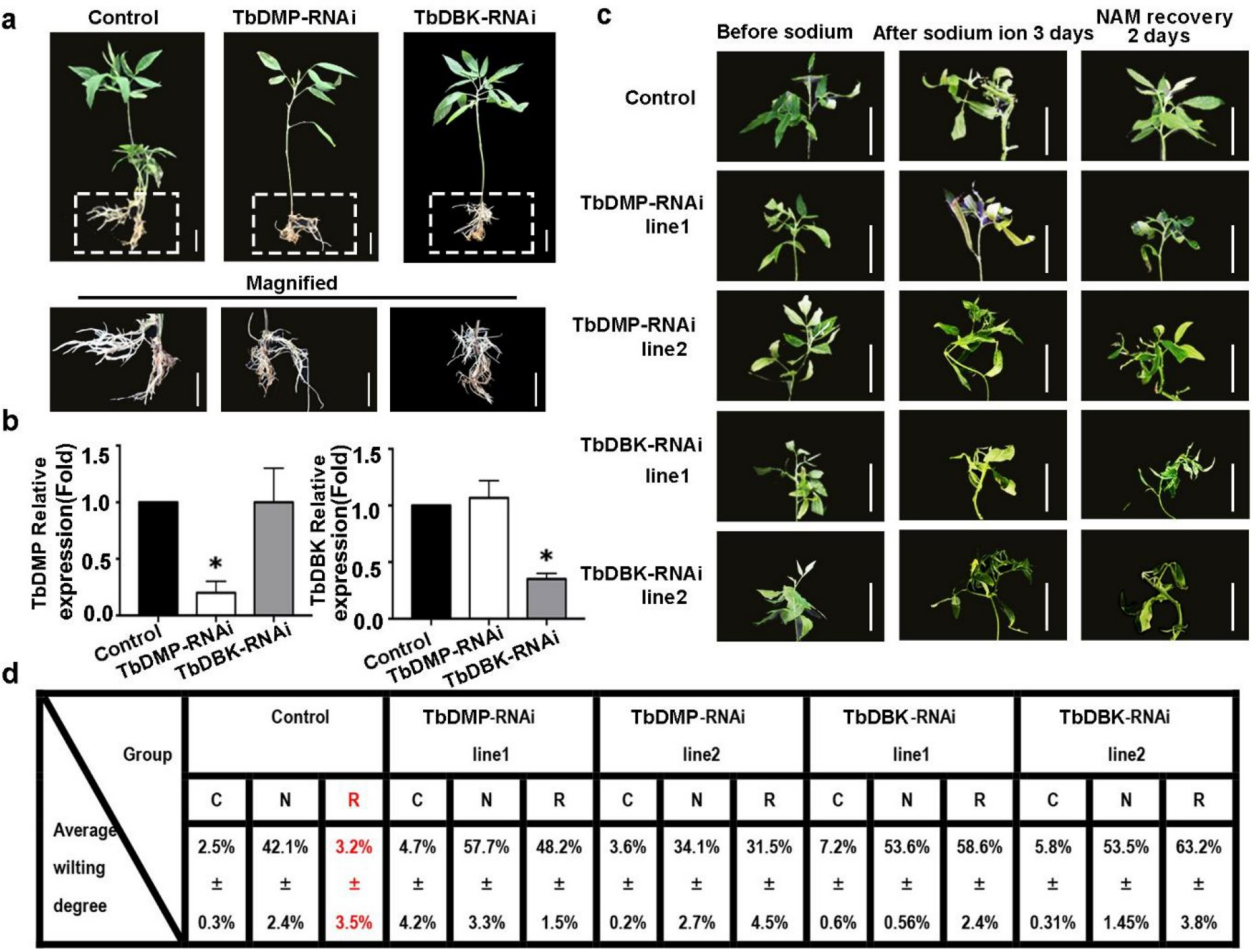
Hairy root transgenic analysis. a. The phenotype of TbDMP-RNAi and TbDBK-RNAi hairy root transgenic lines before and after melatonin treatment. The coding sequences of were cloned into the pROK2 vector and transformed into the *Agrobacterium rhizogenes* stains respectively. b. The relative expression change folds of DMP and TbDBK in transgenic lines. c. The phenotype of DMP and TbDBK transgenic lines before and after 3-days salt treatment. The phenotype of transgenic lines NAM recovery after 2 days was also shown in figure. Bars in a and c is 2 cm. d. Survival rate of transgenic lines in c. Each data point is mean ±SD of three replicates.

Previously, we showned that NAM can significantly alleviate sodium ion stress in tree bean and concomitantly elevate the expression level of TbDMP. Since TbDMP and TbDBK may interact, it is reasonable to suggest that NAM may promote this interaction. To answer this question, we tested the colocalization of TbDMP and TbDBK in samples treated with or without NAM. The TbDMP protein was fused with GFP and the TbDBK protein was fused with m-Cherry and both vectors were transiently expressed in the tobacco leaf cells (Fig 7). A green fluorescence signal was produced by the TbDMP protein, and a red fluorescence signal was emitted by the TbDBK protein. The yellow fluorescence in the superimposed image represents the colocalization region. We found that the colocalization and interaction intensity of the two proteins are apparently increased when NAM was sprayed on the leaves; The fluorescence intensity of the image and statistical fluorescence values were elevated based on an evaluation ImageJ and Origin software results (Fig 7A and 7B). To further analyze the colocalization of the two proteins in tobacco leaf cells, the signals were detected at 360 degrees of three-dimensional space. The results suggest that spraying of NAM can improve the colocalization intensity of the two proteins (Fig 8). Other studies have shown that AtDBK physically interacts with and phosphorylates AtADF4 to inhibit AtADF4 activity in cytoskeleton filament disassembly, which means that the stomatal close primarily through phosphorylation of ADF and are able to resist drought in *Arabidopsis*. In this study, NAM spraying enhanced the interaction between the two proteins. It is possible that NAM alleviates drought stress by enhancing phosphorylation and induces sodium ion stress resistance by inhibiting the depolymerization of cytoskeleton cytoskeleton. TbDMP of tree bean belongs to subclass I (Fig 4A). Therefore, we used the hairy root transgenic system to validate the role of the two proteins in the alleviation of sodium ion stress by NAM. Based on the traditional hairy root method, we previously established an efficient agrobacterium injection system in tree bean seedlings and 11 economically important plants, including *Hibiseu manihot* L (Herb), *Caragana sinica* (shrub), *Malus domestica* gala (tree), etc. (unpublished data). The constructed vector was introduced into the *Agrobacterium rhizogenes* strain and then injected into the half-month-old tree bean seedlings. After approximately 14–21 days, the small hairy roots, which are similar to adventitious roots, grew from the calluses. After at least a month later, the hairy roots grew large enough to provide nutrients to the whole plant and were able to assist in plant development (Fig 9A). TbDMP-RNAi and TbDBK-RNAi vectors were separately transfected into the roots and then the plants were sodium ion-stressed. The data indicate that the spraying of NAM does not completely relieve the stress. In particular, the number of well-arranged microfilaments in the root cells did not increase. The relative expression changes in TbDMP and TbDBK in transgenic lines were effectively detected (Fig 9B). To evaluate the impact of TbDMP and TbDBK RNAi on MT recovery under sodium ion tolerance, we used four-week-old transgenic and control (empty vector) plants in soil. After 3 days of sodium ion stress, four transgenic lines exhibited similar sodium ion tolerance as that in the control plants (Fig 9C and 9D, where C represents the control, N represents NaCl, and R represents recovery). The survival rates were measured after the plants were under NAM recovery and allowed to grow for 2 days. These results indicate that NAM can alleviate sodium ion stress in tree bean. With regard to the root cytoskeleton structure, the data indicate that the filament rearrangement was improved by increasing the TbDMP-TbDBK interaction intensity.

## Discussion

Although a of management methods have been used to overcome sodium ion stress in agricultural production, certain practices are not particularly economical. However, cultivation of sodium ion-tolerant crop varieties that produce economically valuable yields, even when affected by sodium ion soils with high salinity, is a feasible and effective option [28]. sodium ion stress treatment of sunflower seedlings showed that primary root growth and hypocotyl elongation were inhibited; however, when NAM was used in combination with sodium ion, the primary root length and hypocotyl elongation increased by 13% and 58%, respectively. In a study of rooting of sweet cherry rootstocks, a low concentration of NAM promoted root growth but a high concentration of NAM inhibited root growth. Another study demonstrated that the effect of NAM on adventitious root formation in tomato seedlings is dose-dependent [29]. In the present study, the expression level and interaction intensity were upregulated and enhanced by NAM to resist sodium ion stress. After NAM treatment, the *CBL1* and *CIPK23* genes were significantly upregulated in the sodium ion-sensitive transgenic plants in addition to SOS1, SOS2, SOS3, and *NHX*s, activated the SOS pathway and improved plant sodium ion tolerance [30]. Therefore, we hypothesized that sodium ion stress in tree bean can be resisted via effects on the microfilament cytoskeleton.

TbDBK is related to resistance to drought stress in *Arabidopsis thaliana* and is sensitive to abscisic acid (ABA) treatment and is involved in ABA and drought-induced stomatal closure. During physiological stomata closure, TbDBK physically interacts with and phosphorylates DMP thus inhibiting its activity in cytoskeleton disassembly [31]. After stomatal closure, TbDBK can inhibit cytoskeleton filament disassembly and ultimately resist drought. NAM may interact with other plant hormones because numerous genes are influenced by NAM, which also participates in other hormone signaling pathways, such as ABA, SA, auxin, ETH and JA [32]. The common feature of these studies is that the content of ABA is significantly decreased after NAM treatment. Certain studies have shown that NAM at 100 μM can selectively down-regulate MDNCED3 and up-regulate MDCY707A1 and MDCY707A2 in apple under drought stress resulting in a decrease in ABA content in the apple plants under drought stress [33]. The ABA biosynthesis and the signal genes of perennial ryegrass were significantly down-regulated by 20 μM NAM treatment to alleviate heat stress [34]. The antistress effects of NAM may involve various genes downstream of the ABA signal and skeleton dynamics thus establishing a new network of hormone signaling and stress. These results suggest that NAM can regulate the ABA signal transduction pathway; however, the specific mechanism underlying this regulation is more complex and involves a number of genes and pathways. Thus, additional studies are needed.

In this study, NAM enhanced the interaction intensity of the TbDMP-TbDBK protein complexes; the mechanism of NAM-induced resistance to sodium ion stress is unclear. In previous studies, the cytoskeleton disassembling activity of TbDMP was enhanced by various factors, such as increased intracellular pH. The cytoskeleton disassembling activity of DMP is decreased by the phosphoinositide and by phosphorylation of DMP [16, 35].

Several studies described casein kinase regulation of microfilament-binding proteins that resist adversity in lower and higher eukaryotes. For example, in yeasts, hrr25 of kinase CKL1 can phosphorylate a number of ABP including cofilin 1. Casein kinase 1 is a highly conserved serine/threonine protein kinase family in eukaryotic organisms and a number of cytoskeleton-related proteins have been identified as its targets. These targets include cofilin, twinfilin, myosin, tropin, spectrin 3, dynein, microtubule-binding protein and motor protein-like protein [36]. In *Arabidopsis thalian*a, CKL6 can interact with and phosphorylate tubulin, thereby ultimately regulating tissue microtubules in the process of plant development [36]. Under drought stress and ABA treatment, TbDBK and cytoskeleton protein 4 participate in stomatal closure, which is also due to the physical interaction between TbDBK and cytoskeleton protein 4 and the phosphorylation of cytoskeleton protein 4 by TbDBK, which eventually reduces the cytoskeleton depolymerization activity of cytoskeleton protein 4 *in vitro*, leading to stomatal closure and drought resistance.

## Supporting information

S1 Fig Moderate amount of NAM can alleviate root growth inhibition and fresh weight reduction caused by sodium ion treatment. 5-day-old and 2-week-old tree bean seedlings were treated with different sodium ion concentrations (0 20 40 80 and 100 mM NaCl) for 3 days. Average root length **(a)** and fresh weight **(b)** were measured to determine a suitable sodium ion concentration for following NAM treatment. Sodium ion treated seedlings were grown on various NAM levels (0, 50, 100, 200, 400μM) to determine an appropriate level of melatonin for sodium ion stress relief **(c)**.
(DOCX)

S1 Table The acronyms list. (XLSX)

S2 Table The primers used in this study. (XLSX)

S3 Table Positive clone obtained during Y2H screening by TbDMP. (XLSX)
