# Supplementary material for: Role of a complex of two proteins in alleviating sodium ion stress in an economic crop
Source: PLoS One. 2020 Nov 20;15(11):e0242221. doi: 10.1371/journal.pone.0242221 (PMC7679020; doi:10.1371/journal.pone.0242221)
Supplement: S1 Fig — 5-day-old and 2-week-old tree bean seedlings were treated with different sodium ion concentrations (0 20 40 80 and 100 mM NaCl) for 3 days. Average root length (a) and fresh weight (b) were measured to determine a suitable sodium ion concentration for following NAM treatment. Sodium ion treated seedlings were grown on various NAM levels (0, 50, 100, 200, 400μM) to determine an appropriate level of melatonin for sodium ion stress relief (c). (DOCX) [file pone.0242221.s001.docx]

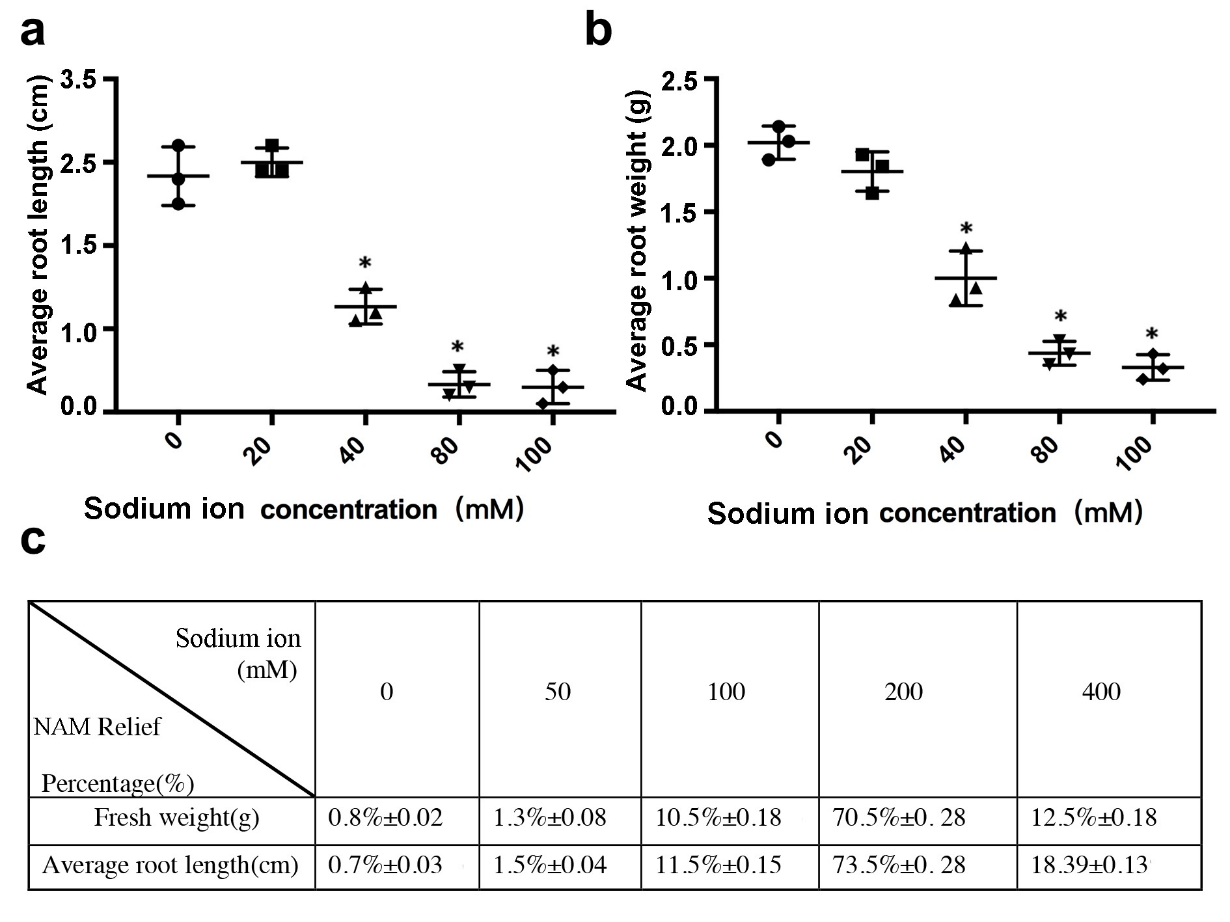


**Figure S1. Moderate amount of NAM can alleviate root growth inhibition and fresh weight reduction caused by sodium ion treatment.**

5-day-old and 2-week-old tree bean seedlings were treated with different sodium ion concentrations (0 20 40 80 and 100 mM NaCl) for 3 days. Average root length **(a)** and fresh weight **(b)** were measured to determine a suitable sodium ion concentration for following NAM treatment. Sodium ion treated seedlings were grown on various NAM levels (0, 50, 100, 200, 400μM) to determine an appropriate level of melatonin for sodium ion stress relief **(c)**.
